# Identification of the p34 Protein of African Swine Fever Virus as a Novel Viral Antigen with Protection Potential

**DOI:** 10.3390/v16010038

**Published:** 2023-12-25

**Authors:** Xin Zhang, Xiangyu Guan, Qiuxia Wang, Xiao Wang, Xiaoke Yang, Shuwen Li, Xiao-Tian Zhao, Mengqi Yuan, Xingyou Liu, Hua-Ji Qiu, Yongfeng Li

**Affiliations:** 1 State Key Laboratory for Animal Disease Control and Prevention, Harbin Veterinary Research Institute, Chinese Academy of Agricultural Sciences, Harbin 150069, China; m18317204106@163.com (X.Z.); gxy15054837089@163.com (X.G.); m15225539379@163.com (X.Y.); lishuwen176@163.com (S.L.); alezxt@163.com (X.-T.Z.); yuanmengqi2019@163.com (M.Y.); 2 College of Animal Science and Veterinary Medicine, Henan Institute of Science and Technology, Xinxiang 453003, China; wqxmjz@126.com (Q.W.); lxingyou@sohu.com (X.L.); 3 Department of Pathogenic Biology, School of Basic Medical Sciences, Binzhou Medical University, Yantai 264003, China; wangxiao2037@126.com

**Keywords:** African swine fever virus, p34 protein, antigenicity, humoral and cellular immunity

## Abstract

African swine fever (ASF) is a highly contagious disease caused by African swine fever virus (ASFV), affecting domestic and wild boars. The polyprotein pp220 of ASFV is responsible for producing the major structural proteins p150, p37, p14, p34, and p5 via proteolytic processing. The p34 protein is the main component of the ASFV core shell. However, the immunologic properties of the p34 protein *in vitro* and *in vivo* remain unclear. The results showed that the recombinant p34 protein expressed in prokaryotes and eukaryotes could react with convalescent swine sera to ASFV, suggesting that p34 is an immunogenic protein. Significantly, anti-p34 antibodies were found to inhibit the replication of ASFV in target cells. Furthermore, rabbits immunized with the recombinant C-strain of classical swine fever virus containing p34 produced both anti-p34 humoral and cellular immune responses. In addition, the p34 protein could induce a cell-mediated immune response, and a T-cell epitope on the p34 protein was identified using immunoinformatics and enzyme-linked immunospot (ELIspot) assay. Our study demonstrates that the p34 protein is a novel antigen of ASFV with protective potential.

## 1. Introduction

African swine fever (ASF) is a hemorrhagic infectious disease caused by African swine fever virus (ASFV) in domestic pigs and wild boars [1,2]. Upon infection with highly virulent strains, clinical signs involve pulmonary edema, high fever, spotty skin, lymphopenia, and hemorrhagic lesions [3]. ASF outbreak was first reported in Kenya in 1921. Although lots of studies on ASF vaccines were reported, there were no commercial vaccines available for ASF prevention and control [4]. The widespread and colossal economic losses from ASF have impacted the development of animal husbandry practices in most countries within Africa, Asia, and Europe [5].

ASFV is a large, enveloped virus with icosahedral morphology and an average diameter of 200 nm [6], and it is a member of the *Asfaviridae* family [7]. ASFV is a large DNA virus with a linear double-stranded genome varying in size from 170 to 194 kb that contains 150 to 167 open reading frames (ORF) [8,9], encodes more than 50 structural and 100 nonstructural proteins, many of which are unessential for viral replication but have roles in interactions with the host to facilitate its survival and transmission. The internalization process of ASFV involves critical steps for infecting host cells [10]. While the precise mechanism of ASFV internalization is still under investigation, several fundamental steps are believed to be involved. ASFV relies on interactions with host cell surface receptors to facilitate internalization [11]. Initial binding typically occurs at the host cell membrane, where the surface proteins of ASFV interact with host cell surface receptors, initiating virus–cell membrane interactions. Subsequently, ASFV can enter host cells through different mechanisms. Viral entry into host cells is a key target for inhibiting ASFV infection [12]. The ASFV particle has an icosahedral morphology composed of several concentric domains: the internal core formed by the central genome contains the nucleoid, which is coated by a thick protein layer named core shell; an inner lipid envelope surrounding the core; and finally, the capsid, which is the outermost layer of the intracellular virions [13]. Since the ASFV p72 can remain genetically stable when other genes of the genome undergo significant changes, more than 20 ASFV genotypes have been reported so far based on the C-terminal sequence of the gene encoding the p72 capsid protein. However, a recent study indicates a reduction of the current 25 genotypes solely based on the p72 sequence down to six distinct genotypes, and it is recommended to replace the previously used Roman numerals with Arabic numerals in order to differentiate between the old and new genotyping systems [14]. The exact functions of most of the structural and non-structural proteins remain unknown.

Despite the fact that several research groups have developed novel vaccine technologies, ranging from inactivated viruses, recombinant proteins/peptides, and DNA vaccines to live-attenuated vaccine (LAV) candidates, an efficacious and safe ASF vaccine does currently not exist [15]. The failure and slowness of ASFV vaccine development is due to the fact that the infection process of the virus is not well defined, and the key protective antigens have not yet been identified. It is well known that the entry of viruses into host cells is receptor-mediated, and the main targets of replication are monocytes and macrophages [16,17,18]. With the development of vaccine research and development technologies, novel vaccines have been widely used in the prevention of various infectious diseases. Due to their desirable safety profiles, some novel vaccines have unique advantages to be applied. The major premise of developing novel vaccines is to screen protective antigens. With the development of various omics research, cutting-edge bioinformatics tools for eukaryotes have been well developed, while the much simpler structure of viruses compared with eukaryotic cells corresponds to relatively simple research methods. Strategies for screening protective antigens need to combine the advantages of both bioinformatics methods and traditional molecular biology methods.

Convalescent swine sera from pigs that have recovered from ASFV infection are a potential treatment option for ASFV. There is currently no effective vaccine or treatment for ASFV. Thus, convalescent swine sera have been explored as a supplementary control measure. The sera contain antibodies against ASFV, which can neutralize the virus and prevent pigs from clinical signs or deaths. When given to infected pigs, convalescent swine sera may help to reduce the severity of the disease and increase the animals’ survival.

The specific portion recognized on an antigen molecule determines its antigenic specificity and is called an antigenic epitope, also known as an antigenic determinant [19]. Antigenic epitope is the part of the antigen that binds to the antibody and is the basis of protein antigenicity. It is the region of the antigen that binds to the antibody and is essential for initiating the cellular immune response in the body [20]. Hence, it is crucial to identify the ASFV-specific T-cell epitopes for the activation of T-cell immune responses.

pp220 is a major polyprotein encoded by the ASFV *CP2475L* gene that is the main component of the core shell of ASFV particles, accounting for one-third of the total proteins of ASFV [21]. It has been demonstrated that the pigs immunized with a combination of adenoviruses expressing the pp220 protein developed significant immune responses, including elevated IgG antibodies, increased IFN-γ-secreting cells, and enhanced cytotoxic T lymphocyte activity [22]. The pp220 polyprotein is initially processed into the p150 protein and the pp90 polyprotein. The latter is cleaved into the p34 protein and the precursor pp55 protein, from which the p5, p14, and p37 proteins are eventually generated. It has been reported that p37 and p14 are localized to the nucleus [23,24]. The structural protein p34 is involved in the formation of the viral replication complex and interacts with other structural proteins, such as p30, to form the virus particle [25]. Studies have shown that mutations in the p34 gene can affect virus replication and assembly [26]. In this study, the immunogenicity of the p34 protein cleaved from the pp220 protein was first analyzed *in vitro* and *in vivo*.

## 2. Materials and Methods

### 2.1. Viruses and Cells

The ASFV HLJ/2018 strain (GenBank no. MK333180.1) was isolated by National African Swine Fever Para-Reference Laboratory, Harbin Veterinary Institute, Chinese Academy of Agricultural Sciences. The CD2v-deleted virus ASFV-ΔCD2v was generated by deleting the CD2v-encoding gene in the backbone of the HLJ/2018 strain. Convalescent swine sera to ASFV were collected in the field. Primary porcine alveolar macrophages (PAMs) were prepared [27] and cultured in RPMI 1640 medium (catalog no. C11875500BT; Gibco, Thermo Fisher Scientific; Waltham, MA, USA) supplemented with 10% fetal bovine sera (FBS) (catalog no. 10091148; Gibco, Thermo Fisher Scientific; Waltham, MA, USA) and 2% antibiotics-antimycotics (catalog no. 15140122; Gibco, Thermo Fisher Scientific; Waltham, MA, USA). HEK293T, SK6 cells were cultured in Dulbecco’s modified Eagle’s medium (DMEM) (catalog no. D6429; Sigma-Aldrich/Merck; Darmstadt, Germany) with 10% FBS at 37 °C with 5% CO_2_.

### 2.2. Sequence Analysis

The p34-encoding sequence of the ASFV HLJ/2018 strain was compared with those of the ASFV Armenia 07, BA71V, Georgia 2007, Malawi Lil 20/2, OUR T88-3, and Wuhan 2019 strains by using the MegAlign software (Version: 7.0).

### 2.3. Construction of the Prokaryotic and Eukaryotic Plasmids Expressing the ASFV p34 Protein

To construct a plasmid expressing the p34 protein, the genome of the ASFV HLJ/2018 strain was used as template to amplify the p34-encoding gene by PCR with the specific primers pET-34-F/R described in Table 1 under the following reaction conditions: 95 °C 5 min, 98 °C 15 s, 56 °C 30 s, and 72 °C 70 s, a total of 30 cycles. Then, the amplified fragment was cloned into pET-28a cut by *Eco*RI and *Xho*I, creating pET-p34.

To construct a recombinant plasmid for eukaryotic expression of the p34 protein, pET-p34 was used as template to amplify the p34-encoding gene by PCR with the primers pShuttle-p34-F/R. The PCR production amplicons were cloned into the pShuttle-CMV vector by using *Kpn*I and *Not*I, creating pShuttle-p34. 

### 2.4. Expression and Purification of the Recombinant p34 Protein

pET-p34 was transformed into *E. coli* BL21(DE3) competent cells using the heat shock method. The bacterial culture was induced with 0.1 mM isopropylthiogalactoside (IPTG) for 4, 8, 16, or 26 h at 37 °C to achieve optimal expression. The recombinant bacteria were cultured, the precipitates were resuspended in PBS and lysed by ultrasound, and the supernatants were collected by centrifugation. According to the requirements, high-affinity Ni^+^-charged resin FF was used to carry out nickel column affinity chromatography on the lysis supernatant under non-deformation conditions, and the eluent containing 200 or 500 mM imidazole was used to elute the target protein. The concentration of the purified recombinant p34 protein was determined by BCA kit. The purified protein was subjected to Coomassie brilliant blue staining and Western blotting.

### 2.5. Antigenic Analysis of p34

To analyze the antigenicity of the p34 protein, the purified recombinant p34 protein was subjected to Western blotting using convalescent swine sera to ASFV as the primary antibody and M800-labeled goat anti-pig IgG as the secondary antibody. The ASFV p54 protein was included as a positive control. In addition, HEK293T cells were transfected with pShuttle-p34 using X-tremeGENE HP DNA transfection reagent. At 48 h post-transfection, the transfected HEK293T cells were detected using indirect immunofluorescence assay (IFA) with convalescent swine sera to ASFV as the first antibody and FITC-labeled goat anti-pig IgG as the second antibody.

### 2.6. The Cell-Mediated Immune Response Induced by p34

As cellular immune response plays an important role in providing immune protection against ASFV challenge, we assessed the cellular immune response activated by the p34 protein. In particular, we determined the ASFV-specific IFN-γ cell response using the ELIspot assay, which involved isolating peripheral blood mononuclear cells (PBMCs) from convalescent pigs. 

### 2.7. Preparation of Anti-p34 Antibodies in Mice

To prepare anti-p34 antibodies, we diluted 50 μg of purified p34 protein with PBS to a total volume of 3 mL and mixed it with Freund’s incomplete adjuvant. We inoculated five-week-old BALB/c mice with the mixture, while a control group was inoculated with PBS alone. Immunization was conducted at two-week intervals, with Freund’s complete adjuvant replacing the incomplete adjuvant during the third immunization. One week after the third immunization, we collected blood samples from the mice by eyeball extraction and separated the sera.

### 2.8. Generation of a Recombinant HCLV Expressing p34

To further verify the immunogenicity of the p34 protein, we constructed a recombinant HCLV that expresses the p34 protein. We used the primers listed in Table 1 to construct this virus, with the p34-encoding gene inserted and expressed as a separately expressed form between the N^pro^ protein and the C protein. We then identified the recombinant virus using RT-PCR and indirect fluorescent assay (IFA) (Figure S1) and analyzed its growth curve and genetic stability (Figure S2).

### 2.9. Animal Immunization Experiment

Sixteen New Zealand rabbits of fourteen weeks of age were randomly divided into three groups (Groups A, B, and C). Group A (rabbits #1 to 6) was immunized with 10^4^ TCID_50_ rHCLV, Group B (rabbits #7 to 12) was immunized with 10^4^ TCID_50_ rHCLV-p34 through the auricular vein, and Group C (rabbits #13 to 16) was immunized with 1 mL of DMEM serving as a negative control. The rectal temperature was recorded every 6 h from 24 h post-immunization to determine the fever response of the rabbits (Figure S3A). After the rectal temperature of the rabbits returned to normal, three rabbits in each group were selected and sacrificed. The spleen of the rabbits was collected, and the viral genome copies in the spleen were determined by quantitative real-time RT-PCR (Figure S3B). The remaining rabbits were immunized with the same dose of the corresponding virus at 14 and 28 days post-immunization (dpi). Serum samples were collected every 7 days after immunization. Whole blood samples were also taken from all the rabbits on these points for the collection of PBMCs.

### 2.10. Detection of the Serum Anti-p34 Antibodies in Rabbits 

PAMs were cultured overnight in 96-well plates in a 37 °C incubator with 5% CO_2_. ASFV-WT was then diluted to 100 TCID_50_/50 μL and added to the plates for 48 h. The cells were repeatedly washed with PBS twice, fixed with 4% paraformaldehyde solution at 20 °C for 15 min, and then treated with 2% Triton X-100. Different dilutions of rabbit serum were used as the primary antibody and added to the cells, which were then incubated at 37 °C for 2 h. The plates were washed and further incubated at 37 °C for 1 h with anti-rabbit IgG conjugated with FITC (Sigma-Aldrich) at a 1:200 dilution with 5% BSA. After washing four times with PBST and once with PBS, 50% glycerol was added to each well. The results were observed and recorded under a fluorescence microscope (Nikon TE200; Tokyo, Japan).

### 2.11. ELIspot Assay

Antigen-specific IFN-*γ*-secreting cells were quantified by an ELIspot assay using a Mabtech kit (catalog no. 3110-4HPW-2; Mabtech; Stockholm, Swedish) according to the manufacturer’s protocols. Briefly, two weeks after the final immunization, whole blood was collected into EDTA-treated vacutainer tubes to isolate PBMCs by density-gradient centrifugation. For PBMC cultures, RPMI 1640 medium was supplemented with 10% FBS (Invitrogen; Carlsbad, CA, USA), 50,000 IU penicillin/L (Invitrogen; Carlsbad, CA, USA), and 50 mg/L streptomycin (Invitrogen; Carlsbad, CA, USA). The PBMCs resuspended in complete RPMI 1640 media were added to the wells of MultiScreen-HA plates (Millipore/Merck; Darmstadt, Germany) at a density of 2.5 × 10^5^ cells/well. Affinity-purified antigens (final concentration of 2.5 μg/mL) were added to the cells in triplicates. Phytohemagglutinin (PHA) mitogen (final concentration 5 μg/mL) was included as a positive control, with media only serving as a negative control. The spots were counted using an ELIspot reader and AID software (AutoImmun Diagnostica V3.4, Strassberg, Germany). The mean number of IFN-*γ* spot-forming cells (SFCs) for each sample was calculated by subtracting the mean number of spots in the negative control wells from the mean number of spots in the sample wells. The data were presented as the mean number of SFCs per 10^6^ PBMCs.

### 2.12. Serum Neutralization Test

Blood samples were collected from the immunized mice and rabbits in untreated vacutainer tubes 14 days after the last booster immunization, to obtain sera. PAMs were cultured in 96-well plates overnight in a 37 °C incubator with 5% CO_2_. ASFV-ΔCD2v and ASFV-WT were each diluted to 100 TCID_50_ per well. Sera from rabbits immunized with rHCLV-p34 and mice immunized with p34 protein were diluted at 1:5, 1:10, and 1:20 in RPMI 1640 medium, mixed with ASFV-ΔCD2v and ASFV-WT, and incubated for 1 h at 37 °C. The mixture was then inoculated into the PAMs and incubated at 37 °C in 5% CO_2_. At 48 h post-infection (hpi), the infected cells were observed under a fluorescence microscope (Nikon TE200; Tokyo, Japan). The infected cells were lysed, the supernatants were collected to extract DNA as described previously [28], and viral genome copies in the rabbit sera were quantified using qPCR.

### 2.13. Screen of the T-Cell Epitopes on p34

To identify T-cell epitope on the ASFV p34 protein, we synthesized and screened a peptide, IADAINQEF, on the p34 protein. The major histocompatibility complex (MHC) is a glycoprotein located on the cell surface that recognizes antigens and regulates immune function. Porcine MHC is referred to as swine leukocyte antigen (SLA) and is divided into three types: *SLA-I*, *SLA-II*, and *SLA-III*. The *SLA-I* gene is primarily responsible for presenting endogenous antigenic peptides and triggering cellular immunity. The peptides were synthesized by GenScript Biotech Co., Ltd. (Nanjing, China), dissolved in solvents as recommended by the manufacturer, and had a purity of 95% or greater. We also tested the cellular immune response activated by the peptide.

### 2.14. Multiple Sequence Analysis of the Identified Epitope 

The conservatism of epitope amino acid sequences in different strains is crucial for their potential as vaccine candidates. To analyze the conservation of identified linear epitopes in various ASFV strains, eight representative ASFV strains were selected from the NCBI database for sequence comparison. A multi-sequence alignment was performed using MegAlign software (Version: 7.0) and generated an image using ESPript 3.0: https://espript.ibcp.fr/ESPript/cgi-bin/ESPript.cgi (accessed on 10 October 2022).

### 2.15. In Silico Validation of the Candidate Epitopes

SLA-1*0401 PDB crystal structure was utilized for the validation of the candidate epitopes in this study due to the unavailability of crystal structures of other SLAs identified in the study of the RCSB protein data bank to date. Candidate epitopes that bind to SLA-1*0401 were validated by molecular docking. The SLA-1*0401 (PDB 3QQ3) crystal structure was downloaded from the PubMed protein database. To allow docking of candidate epitopes, the influenza epitope initially bound to the influenza-SLA-1*0401 complex was removed manually, and each candidate epitope was docked to SLA-1*0401 in the GalaxyPepDock server. For each type of resulting complex, docked structures with the highest score were chosen and retrieved. The docked structures were then refined using the RefineComplex tool. 

To evaluate the SLA-epitope complex formation stability and flexibility, a molecular dynamics simulation was conducted. The simulation was conducted through the OpenMM toolkit on the Google Colab framework to acquire the plot of the root mean square deviation (RMSD) and root mean square fluctuation (RMSF) against time.

### 2.16. Statistical Analysis

Data were analyzed using software GraphPad Prism (version 6.01) (GraphPad Software Inc., San Diego, CA, USA) and expressed as mean ± SD. Differences were considered significant by *p*-value (*, *p* < 0.05; **, *p* < 0.01).

## 3. Results

### 3.1. The p34 Protein Is a Novel Antigenic Protein of ASFV

**Figure 1:**
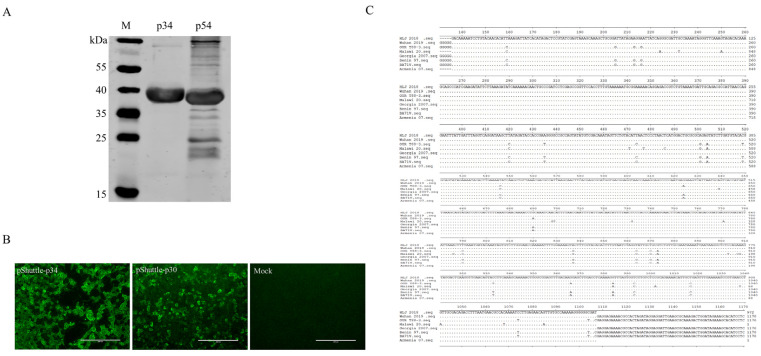
p34 is identified as a novel antigenic protein of ASFV. (**A**) Western blotting was performed on the p34 protein using convalescent swine sera to ASFV. The recombinant ASFV p54 antigen served as a positive control. (**B**) The eukaryotic expression plasmids pShuttle-p34 and pShuttle-p30 were constructed and transfected into HEK293T cells, with pShuttle-p30 used as a positive control. (**C**) The amino acid sequence of p34 is highly conserved among ASFV strains. The p34-encoding sequence of the ASFV HLJ/2018 strain was compared with the ASFV Armenia 07, BA71V, Georgia 2007, Malawi Lil 20/2, OUR T88-3, and Wuhan 2019 strains by using the MegAlign software (Version: 7.0).

To determine the expression of the recombinant p34 protein in prokaryotic cells, Western blotting was performed using convalescent swine sera to ASFV as the primary antibody. The ASFV p54 protein was used as a positive control. The results demonstrated that the recombinant p34 protein could react with the convalescent swine sera to ASFV, indicating that p34 is a new immunogenic protein (Figure 1A).

To further identify the antigenicity of the p34 protein, the eukaryotic expression plasmid pShuttle-p34 was constructed and transfected into HEK293T cells. Specific fluorescence could be observed at 48 hpi by IFA (Figure 1B), indicating that the p34 protein has a strong immunogenicity.

Sequence alignment indicated that the amino acid sequence of the p34 protein is highly conserved among ASFV strains, and the homology is between 96.6% and 100%, indicating that the p34 protein is highly conserved among ASFV strains (Figure 1C).

### 3.2. Anti-p34 Antibodies can Significantly Inhibit ASFV Replication

To evaluate the immunogenicity of rHCLV-p34 in rabbits, different groups of rabbits were inoculated with the indicated viruses, and anti-ASFV p34 antibodies in sera were examined at different time points post-inoculation. The results showed that rabbits in groups A and B were able to induce high levels of anti-E2 antibodies at 7 dpi. With the increase in the number of enhanced immunizations, the levels of anti-E2 antibodies gradually increased. The results of IFA showed that the recombinant virus induced antibodies against p34 in rabbits at 7 dpi (Table 2).

**Figure 2:**
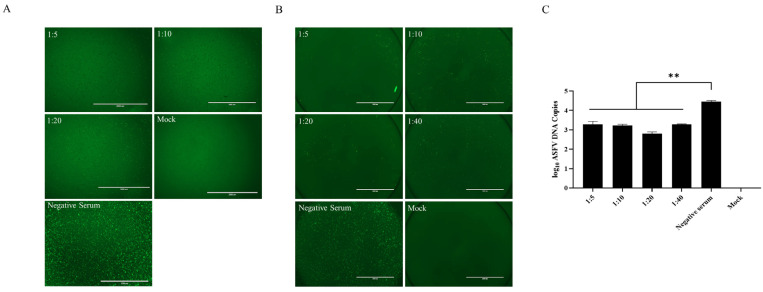
Anti-p34 antibodies inhibit ASFV replication. (**A**) PAMs were infected with ASFV-ΔCD2v mixed with the anti-p34 antisera diluted at 1:5, 1:10, or 1:20, and the fluorescence was observed at 48 hpi. (**B**) PAMs were infected with ASFV-ΔCD2v mixed with the rabbit antisera diluted at 1:5, 1:10, or 1:20, and the fluorescence was observed at 48 h post-infection (hpi). (**C**) ASFV was incubated with the rabbit antisera and the control sera, DNA was extracted at 48 hpi, and viral genome copies were quantified by qPCR. ** *p* < 0.01.

In order to further study its immunogenicity, mice were immunized with the purified p34 protein to prepare anti-p34 antisera to analyze their potential to neutralize the virus. PAMs were infected with ASFV-ΔCD2v mixed with anti-p34 antisera at different dilutions, and the fluorescence was observed 48 h later. The results revealed that anti-p34 antisera with different dilutions could significantly reduce the replication of ASFV-ΔCD2v (Figure 2A), indicating that polyclonal antibodies against p34 can significantly inhibit virus replication.

PAMs were infected with ASFV-ΔCD2v mixed with the rabbit immunized with rHCLV-p34 at different dilution ratios, and the fluorescence was observed 48 h later. The results revealed that the rabbit sera with different dilutions could significantly reduce the replication of ASFV-ΔCD2v (Figure 2B). PAMs were infected with ASFV-WT mixed with the rabbit immunized with rHCLV-p34 at different dilution ratios, and DNA was extracted at 48 hpi for the assessment of viral copies. The measurement of viral genome copies by quantitative PCR (qPCR) showed a 1.7-log10 reduction in viral genome copies in the rabbit sera compared with the control sera (Figure 2C).

### 3.3. p34 Is Able to Activate a Cell-Mediated Immune Response

**Figure 3:**
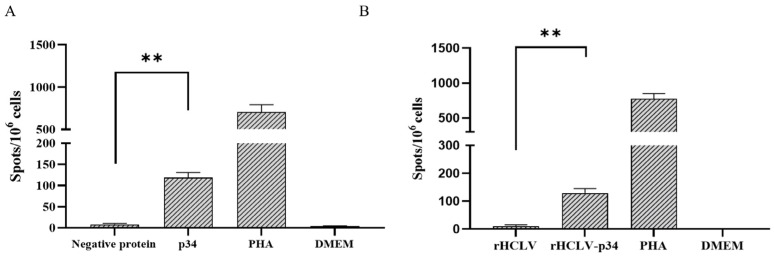
IFN-*γ* response in the PBMCs from the convalescent pigs or the rHCLV-p34-vaccinated rabbits. (**A**) ELIspot assay using the PBMCs from convalescent pigs. The PBMCs collected from the pigs were plated in duplicate and stimulated with DMEM, PHA, the p34 protein, and a negative protein. (**B**) The PBMCs from all the experimentally infected rabbits were stimulated with the p34 protein and evaluated by the ELIspot assay. Mock and PHA stimulation were also carried out as negative and positive controls, respectively. The results were presented as spot-forming cells (SFCs)/10^6^ PBMCs or splenocytes, and the data shown were minus media background counts. Mean responses for each group are indicated by bars, and statistically significant differences between groups are denoted by asterisks. ** *p* < 0.01.

Since cellular immune response plays an important role in the immune protection against ASFV challenge, the cellular immunity response activated by the p34 protein was tested. The ASFV-specific IFN-*γ* cell response was quantified by ELIspot assay using the PBMCs isolated from the convalescent pigs. The cells producing IFN-*γ* were detected upon stimulation with the p34 protein (Figure 3A), suggesting that p34 is able to induce a cell-mediated immune response.

The IFN-*γ* response was examined in the PBMCs from the vaccinated rabbits using ELIspot, and all the group B rabbits developed IFN-*γ*-producing cells (Figure 3B), while the PBMCs from the groups A and C animals remained unresponsive to the virus stimulation.

### 3.4. A T-Cell Epitope Is Present on the p34 Protein

**Figure 4:**
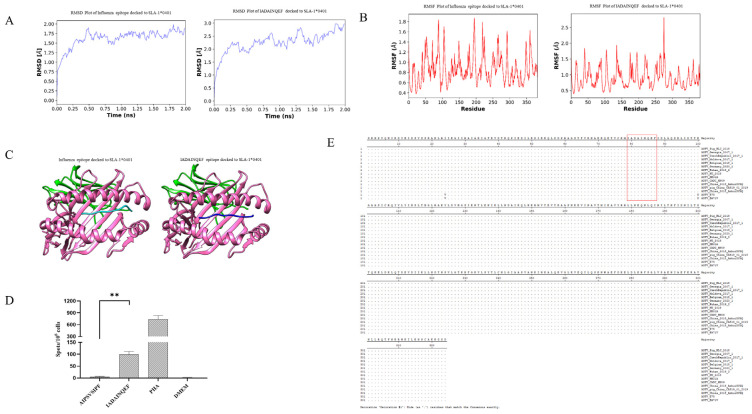
A T-cell epitope is present on the p34 protein. The root mean square deviation (RMSD) and root mean square fluctuation (RMSF) were used to detect the binding of the epitope with SLA-1*0401. (**A**) Root-mean-square deviation plots of the positive reference and the CD8^+^ T-cell epitope IADAINQEF for the residues of swine leukocyte antigen–epitope complexes under 5-ns simulation. (**B**) Root-mean-square fluctuation plots of the positive reference and the CD8^+^ T-cell epitope IADAINQEF for the residues of swine leukocyte antigen–epitope complexes under 5-ns simulation. (**C**) The 3D complex structures of positive reference and the CD8^+^ T-cell epitope IADAINQEF were docked to the binding groove of swine leukocyte antigen-1*0401. (**D**) The major histocompatibility complex (MHC) restriction for stimulation of T cells by synthetic epitopes was identified as MHC class I. Further experiments showed that IFN-*γ* secretion was detected upon *in vitro* restimulation with a peptide by ELIspot. (**E**) Multiple sequence alignment of the epitope of ASFV of different genotypes. ** *p* < 0.01.

Each epitope that binds to SLA-1*0401 was docked to SLA-1*0401, which includes a predicted binding epitope of p34: IADAINQEF. The NSDTVGWSW motif, an influenza epitope that binds to SLA-1*0401, was used as a positive control in this study, and the dissociation constant (*K*_d_) and the binding energy (∆G_bind_) per SLA-epitope complex were used to assess the potential of the epitopes to bind to SLA-1*0401 as shown in Table 3. The results show that the *K*_d_ and ∆G_bind_ values are found to be comparable to that of the value for the influenza epitope-SLA complex, and the binding of epitopes to SLA-1*0401 is spontaneous and stable. Next, to evaluate the SLA-epitope complex formation stability and flexibility, a molecular dynamics simulation was conducted. Furthermore, the epitope-SLA complex stability and flexibility were also validated by the OpenMM toolkit on the Google Colab framework to acquire the plot of the root mean square deviation (RMSD) and root mean square fluctuation (RMSF) against time, which showed that the RMSD and RMSF of the influenza epitope reference and SLA-1*0401 complexes (Figure 4A,B). The RMSD values generated for each complex ranged from 1.0 to 3.0 Å, and the RMSF values generated were less than 4.0 Å. The results showed that the combination of this epitope bound to SLA-1*0401. The 3D complex structures of the candidate and control epitopes, along with the binding groove of the swine MHC-I, were shown in Figure 4C.

To identify the T-cell epitope on the ASFV p34 protein, we synthesized and screened a peptide, IADAINQEF, on the p34 protein. The results showed that IFN-*γ-*secreting cells were detected upon *in vitro* restimulation with the peptide using ELIspot (Figure 4D). Furthermore, sequence analysis of the epitope demonstrated that the sequence of the epitope was highly conserved in different genotypes of virus strains (Figure 4E). 

## 4. Discussion

Since the outbreak of ASF was first reported in Kenya in 1921, ASF has been epidemic for more than one hundred years. ASF is characterized by hemorrhage and high mortality, resulting in huge economic losses in the global pig industry. Despite the recent advances in ASF vaccine development [29], there are no licensed ASF vaccines. Inactivated ASFV vaccines have failed to confer protection against ASFV infection [30]. Some live attenuated vaccines against ASF still suffer from severe side effects and safety issues [31]. In contrast, virus-vectored vaccines of ASFV have particular advantages with regard to safety, and it is most important to choose a suitable presenting antigen. The antigenicity of the p34 protein, as one of the hydrolyzed products of the ASFV polyprotein pp220, remains unknown. It has been reported that cellular immune responses significantly contribute to the protective immunity against intracellular ASFV infections [32,33]. In this study, the recombinant p34 expressed in prokaryotic or eukaryotic cells can react specifically with the convalescent swine sera to ASFV. Furthermore, anti-p34 antibodies could significantly inhibit the replication of ASFV in the target cells, and the p34 protein could activate cellular immunity. And the rabbits immunized with the recombinant CSFV C-strain harboring p34 produced ASFV-specific humoral and cellular immune responses.

Developing a safe and effective ASF vaccine to ensure the supply of pigs is an urgent problem to be solved in pig breeding. Both cellular and humoral immunity plays an important role in anti-ASFV infection. ASFV infection usually results in a mortality rate of nearly 100% in domestic pigs, but pigs survive infection with lower virulence ASFV isolates have a protective immunity against ASFV. Humoral immune responses are important to protect against ASFV infection [34,35]. Anti-ASFV antibodies implicated in neutralization may protect pigs against lethal virus infection. However, knowledge of the immune components associated with protection is very limited. The identification of ASFV antigens responsible for inducing these protective antibodies is of great relevance for vaccine development against this disease. ASFV proteins, such as p72, CD2v, p12, p54, D117L, and p30, can induce neutralization antibodies, in which p72 and p54 can inhibit virus adsorption, p72 and p30 can activate CTL responses, p30 can inhibit virus internalization, and the membrane proteins CD2v, p12, and D117L are involved in inhibiting virus invasion and release [36]. A considerable number of previous studies have endeavored to develop various types of ASF vaccines. The traditional inactivated vaccines against ASF can induce a high level of specific antibodies but cannot provide effective protection against virulent challenges, while attenuated vaccines have safety problems in pigs [37]. However, several studies have found that the antibodies against major structural proteins, such as p30, p54, and p72, are insufficient to provide antibody-mediated protection [28]. Additionally, Marek et al. showed that antibodies against ASFV cannot effectively inhibit ASFV replication *in vitro* [38], suggesting that both effective humoral and cellular immune responses are involved in the protection from virulent ASFV challenge. Through our previous research and the findings of this study, it has been observed that the p34 protein exhibits a strong immunogenicity. Although immunization and challenge trials have not yet been conducted in pigs, this protein is reactive with the convalescent swine sera to ASFV. Furthermore, anti-p34 antibodies effectively inhibit the replication of ASFV, which warrants further evaluation of the p34-based vaccine candidate in pigs. Considering that a single antigen might not be sufficient to induce protective immunity, we plan to assess multiple antigens in pigs. In order to address the safety concern of the vaccines, nucleic acid vaccines, subunit vaccines, and virus-vectored vaccines have been developed. These vaccines mainly choose to present single or multiple immune protective antigens of the virus to induce specific antibodies. CD8^+^ T lymphocytes play important roles in anti-ASFV infection, and T cells, CTL cells, and NK cells are also involved in the protection [39]. Burmakina et al. analyzed the cellular response to ASFV protein in the presence of ASFV infection by ELIspot and identified six T-cell epitopes on CD2v and C-type lectins using synthetic short peptides [40].

In this study, we constructed the recombinant CSFV expressing the ASFV p34 protein based on a reverse genetics system. The genetic stability and growth kinetic characteristics of the recombinant were identified, and its efficacy and safety in animals were preliminarily evaluated. We used the low virulence virus as a vector and inserted the virus protective antigen gene into the genome of the vector. It has been demonstrated that virally vectored vaccines based on baculovirus, adenovirus, and vaccinia virus can effectively elicit both humoral and cellular immunity while providing partial protection against virulent challenge [41,42]. The pigs were immunized with the recombinant adenoviruses expressing eight ASFV proteins (B602L, B646L, CP204L, E183L, E199L, EP153R, F317L, and MGF505-5R) of ASFV and then challenged with 10^4^ HAD_50_ OUR/T88/1 after booster immunization with recombinant poxvirus; all the immunized pigs survived [37]. A study using recombinant adenoviruses expressing 35 ASFV antigens showed that although the immunized pigs could not be protected from virulent challenge [43], a low titer of virus was detected in the tissues of antibody-positive animals, which indicated that the immune response induced by recombinant adenovirus was helpful to inhibit ASFV replication. The detection of IFN-γ-producing cells upon stimulation with the p34 protein indicates that T cells, which are very important for clearing infectious agents, have been activated. This is likely part of the immune response to an infectious agent or other pathogen. Further analysis of the specific immune cells producing IFN-γ in response to the p34 protein could provide important insights into the nature of the immune response, the identity of the pathogen, and the mechanisms underlying the immune response. This information could be useful in developing strategies for preventing or treating infections or other immune-related disorders.

In conclusion, the ASFV p34 protein is a novel antigen with protection potential and can be included in candidate ASF vaccines.

## Figures and Tables

**Table 1:** The primers used in this study.

Primers	Sequences (5′-3′)
pET-p34-F	GGTGGACAGCAAATGGGTCGCGGATCCATGGACAAAAATCCTGTACAACAC
pET-p34-R	GCAAGCTTGTCGACGGAGCTCGAATTCTTAATCGCCCCCCTTTTTGGCACAAC
pShuttle-p34-F	ACCGGCGTGCACTCCGTCGACATGGACAAAAATCCTGTACAAC
pShuttle-p34-R	GGATATCTTATCTAGAAGCTTTTAATCGCCCCCCTTTTTGGCAC
pHCLV-p34-1F	GGAACCGGTGTACGATGCCACGGGGAG
pHCLV-p34-1R	GTGTTGTACAGGATTTTTGTCCATCCCACTTGCGCCATCATCGGA
pHCLV-p34-2F	TCCGATGATGGCGCAAGTGGGATGGACAAAAATCCTGTACAACAC
pHCLV-p34-2R	CAGCAGCGAAAAGTTTGTGGCATCGCCCCCCTTTTTGGCACA
pHCLV-p34-3F	TGTGCCAAAAAGGGGGGCGATGCCACAAACTTTTCGCTGCTG
pHCLV-p34-3R	CTGATGCATGCACCTTGACAGTCGTG

**Table 2:** Immunization protocols and immunogenicity of the recombinant virus in rabbits.

Groups	Viruses	Dose (Boost at Day 14 and 28)	Route	Number of Pigs	Fever Response	Anti-p34 Antibodies	Anti-E2 Antibodies
A	HCLV	10^4^ TCID_50_	i.v.	6	+	-	7 dpi
B	rHCLV-p34	10^4^ TCID_50_	i.v.	6	+	7 dpi	7 dpi
C	DMEM	1 mL	i.v.	4	-	-	-

Notes: Data shown as fever response and viral replication in the rabbits inoculated with the recombinant virus. Anti-E2 antibodies induced by the recombinant virus in the rabbits were detected by ELISA, and anti-p34 antibodies induced by the recombinant virus in the rabbits were tested by IFA.

**Table 3:** Binding energy and binding affinity of SLA-epitope complexes.

Epitopes Docked to SLA-1*0401	ΔG_bind_ (kJ/mol)	*K*_d_ (mol/L)	Antigen Source
IADAINQEF	−9.8	1.3 × 10^−7^	ASFV p34
NSDTVGWSW	−9.8	1.4 × 10^−7^	Positive epitope

## Data Availability

All data are contained in the manuscript, figures, and supplementary figures.

## Supplementary Materials

The following supporting information can be downloaded at: https://www.mdpi.com/article/10.3390/v16010038/s1, Figure S1. Identification of the recombinant CSFV rHCLV-p34 expressing the p34 protein. To further verify the immunogenicity of the p34 protein, we constructed a recombinant CSFV that expresses the p34 protein. We generated the virus harboring the p34-encoding gene between the N^pro^ protein and the C protein. We then identified the recombinant virus rHCLV-p34 using RT-PCR and indirect fluorescent assay (IFA). Figure S2. Characterization of rHCLV-p34. The growth dynamics and genetic stability of rHCLV-p34 were analyzed. Figure S3. The fever response of the rabbits inoculated with rHCLV-p34. The rectal temperature was recorded every 6 h at 24 h post-immunization to determine the fever response of the rabbits. Figure S4. The replication of rHCLV-p34 in the spleens of the inoculated rabbits. When the rectal temperature of the rabbits returned to normal, three rabbits in each group were selected and sacrificed. The spleens of the rabbits were collected and the viral genome copies in the spleens were determined by quantitative real-time RT-PCR. Each of these was conducted with three parallel replicates. Bars show means ± SDs. ns, no significance, *p* > 0.05 (two-way ANOVA).
