# Supplementary figures and images for: Identification of the p34 Protein of African Swine Fever Virus as a Novel Viral Antigen with Protection Potential
Source: Viruses. 2023 Dec 25;16(1):38. doi: 10.3390/v16010038 (PMC10818326; doi:10.3390/v16010038)

Fig. S1

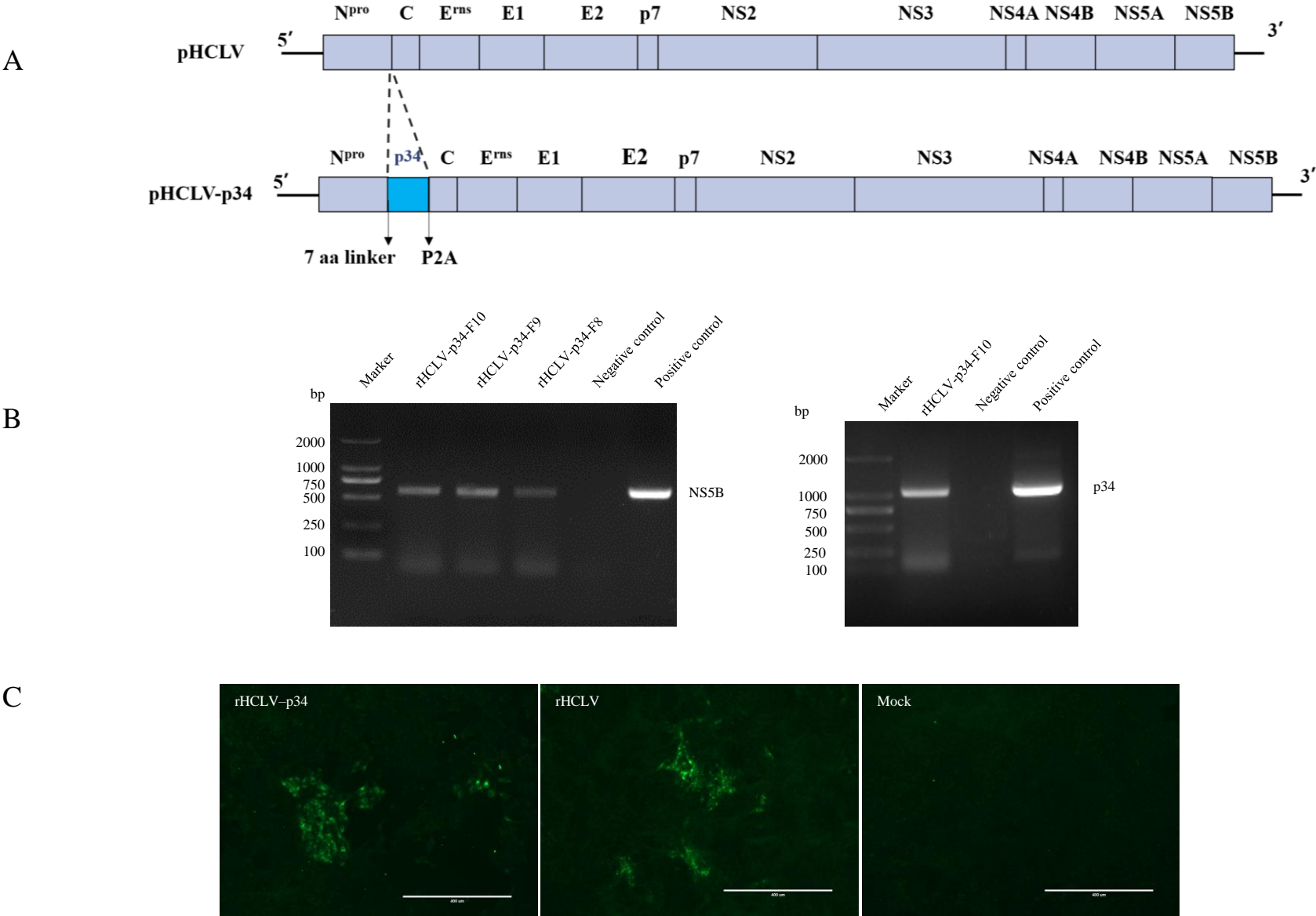

**Fig. S2**

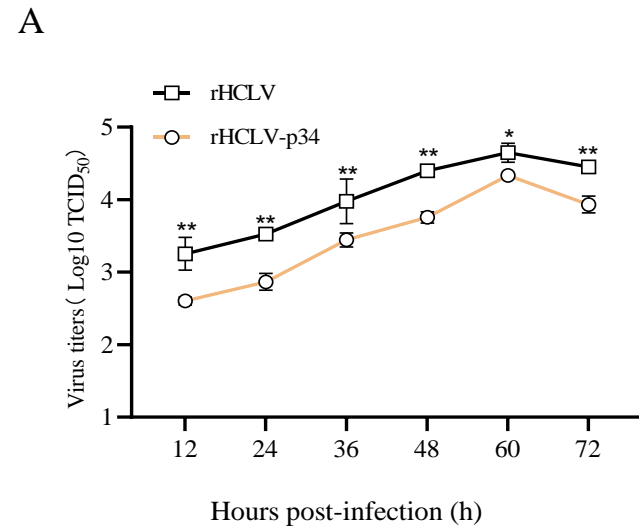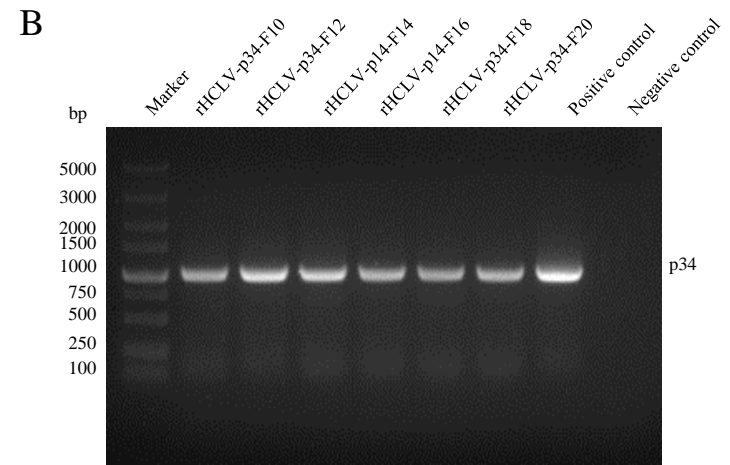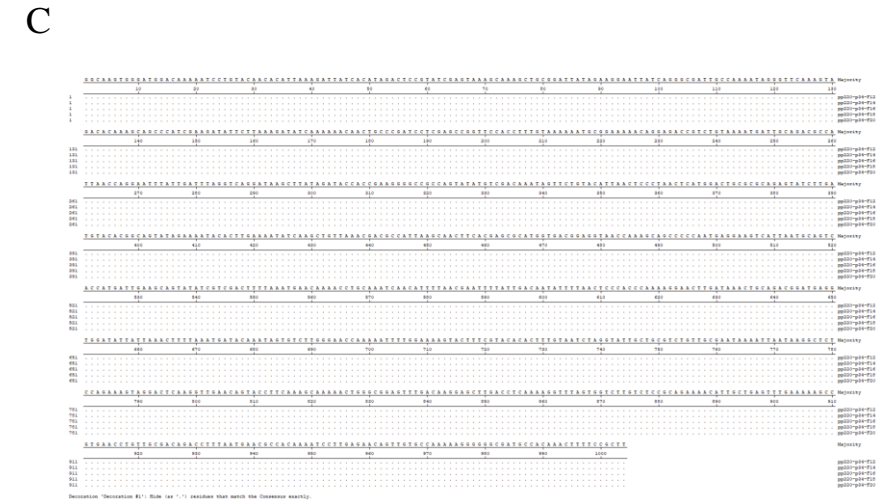

**Fig. S3**

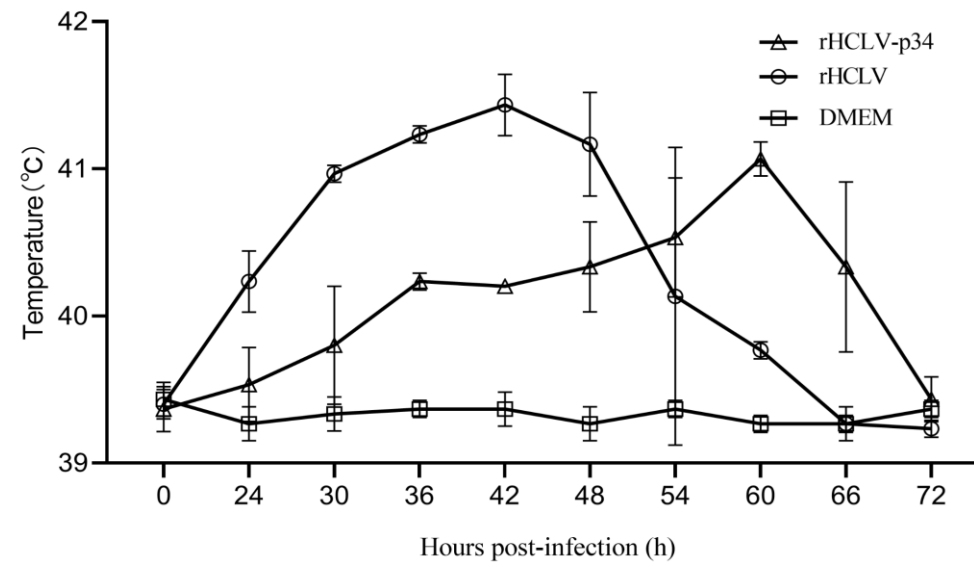

**Fig. S4**

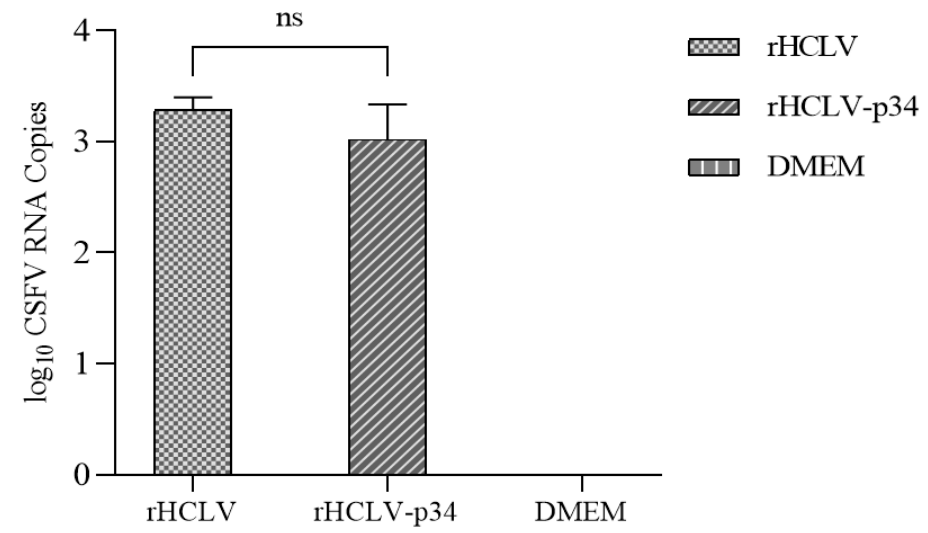

Supplement: Supplementary file 1 [file viruses-16-00038-s001.zip › viruses-2755793-supplementary.pdf]
